# Predicting the prognosis of glioma by pyroptosis‐related signature

**DOI:** 10.1111/jcmm.17061

**Published:** 2021-11-23

**Authors:** Bo Chao, Fenjun Jiang, Huiru Bai, Peipei Meng, Lu Wang, Fei Wang

**Affiliations:** ^1^ Department of Neurosurgery Affiliated Hospital Inner Mongolia Medical University Inner Mongolia China; ^2^ Department of Neurosurgery Sanbo Brain Hospital Capital Medical University Beijing China; ^3^ Basic Medicine college of Inner Mongolia Medical University Inner Mongolia China; ^4^ Academy of Chinese Medical Sciences Guang'anmen Hospital Beijing China; ^5^ School of International Medical Technology Shanghai Sanda University, Guang'anmen Hospital Shanghai China

**Keywords:** glioma, immunity, prognosis, pyroptosis

## Abstract

Glioma is the most common malignant primary brain tumour. It is of great significance for the prognosis and personalized treatment of glioma patients to accurate identification of glioma based on biomarkers. Pyroptosis, a kind of programmed cell death, is closely related to tumour progression and tumour immune microenvironment. However, the role of pyroptosis in glioma remained unclear. Herein, we used glioma clinical and expression data from TCGA and CGGA to explore the relationship between pyroptosis and glioma. We first summarized the incidence of copy number variations and somatic mutations of 33 pyroptosis‐related genes and explored prognostic correlation of these genes. Based on pyroptosis‐related genes, three molecular subgroups of glioma related to prognosis were identified. We also found that each subgroup has unique immune and biological behaviours characteristics. Finally, based on 7 pyroptosis‐related genes (CASP3, CASP4, CASP6, CASP8, CASP9, PRKACA and ELANE), we constructed a prognosis model by Lasso and Cox regression, which had a strong predictive power for the overall survival in CGGA test cohort (*p *< 0.05). In summary, we explored the role of pyroptosis‐related genes in gliomas and the association of these genes with tumour immunity. We found the biomarkers valuable to diagnosis and prognosis, hence, provide reference to the development and treatment of tumorigenesis in glioma.

## 1 INTRODUCTION

Glioma is the most common primary intracranial tumour,^1^ caused by glial or precursor cells. Despite there are already some great advances in molecular targeted therapy,^2^ immunotherapy^3^ and other therapeutic strategies, the overall survival (OS) of glioma has not improved, and a 5‐year OS overall survival rate less than 35%.^4^ Rapid development in molecular biology and genomics^5^, ^6^ has contributed to the discovery of prognostic markers for glioma (such as IDH1/2‐mutation,^7^ and MGMT methylation^8^). However, current prognostic markers such as IDH and NOS are widely presented in glioma of different levels of malignancy, resulting in an insufficient guidance for the prognosis of glioma patients.^9^ In addition, due to the complexity of tumorigenesis and development, the prognosis of cancer patients is related to multiple biological pathways. Therefore, it is necessary to discover new prognostic markers through more extensive bioinformatics analysis.

Pyroptosis is a kind of programmed cell death, which is characterized by gasdermin family protein‐mediated pore formation, cellular lysis and the release of pro‐inflammatory cytokines.^10^ Pyroptosis is dominated and executed by GSDMD and GSDME in the gasdermin superfamily member proteins.^11^, ^12^ GSDMD is regulated by caspase‐1/4/5/11, and GSDME is regulated by caspase‐3, both of which are activated to release the lethal active substances of the N‐ and C‐terminal structural domains and initiate pyroptosis, causing cells gradually swell until the plasma membrane ruptures, and releases a variety of inflammatory factors (IL‐1β, IL‐18, ATP, HMGB1, etc.) at the same time.^13^, ^14^ Recently, it has been shown that GSDMA, GSDMB and GSDMC are also involved in the pyroptosis pathway,^15^ and the invasion level of lung cancer samples with high GSDMD expression is severer.^16^ Besides, chemical drugs such as paclitaxel can induce pyroptosis to inhibit tumour proliferation and metastasis.^17^ Pyroptosis plays an important role in tumour immunity. CD8+ T cells and NK cells can induce pyroptosis through the GSDMB‐granzyme A axis,^18^ and this process can be enhanced by IFN‐γ, while the expression of GSDMD is correlated with CD8+ cell markers, and the cleavage of GSDMD in cytotoxic T lymphocytes is increased.^19^

Previous studies have confirmed that pyroptosis plays an important role in tumorigenesis and tumour immune microenvironment. However, certain function of pyroptosis in glioma remains unclear. We therefore explored the expression levels of pyroptosis‐related genes in glioma through systematic research and discussed the effects of these genes on tumour‐related pathways and tumour immune infiltration, thereby determining the prognostic subtypes of gliomas related to pyroptosis. Finally, we constructed a glioma prognostic model based on the above results and verified it with an external test set.

## 2 MATERIALS AND METHODS

### 2.1 Data sets

We acquired RNA‐seq (Fragment Per Kilobase Million, FPKM) of Lower Grade Glioma(LGG)and Glioblastoma Multiforme (GBM) samples, and their corresponding clinical characteristics data are from The Cancer Genome Atlas (TCGA) and Chinese Glioma Genome Atlas (CGGA). Two data sets contained 670 and 693 patient samples, respectively.

### 2.2 Analysis of copy number and mutation of pyroptosis genes

The 33 pyroptosis‐related genes were gotinitial papers. Perl software (5.34.0) and R (4.0.4) were used for CNV analysis, ‘RCircos’ package was used to get the distribution of pyroptosis‐related genes in chromosomes and ‘maftools’ package was loaded for waterfall chart of gene mutations. Meanwhile, the data of gene expression were normalized into FPKM, and the impacts of single‐gene mutation on the expression levels of other genes were further examined by t test. ‘ggplot2’ package was used to visualize the direction and magnitude of the connection between gene expressions and mutations.

### 2.3 Prognosis analysis of pyroptosis‐related genes

We evaluate the association of pyroptosis‐related genes and prognosis of patients with a cut‐off of FPKM>5. R packages ‘igraph’, ‘psych’, ‘reshape2’ and ‘RColorBrewer’ were used to draw the correlation network of prognosis and pyroptosis‐related genes. We used ‘ConsensusClusterPlus’ package to cluster the glioma based on subtypes and further determined the number of subtypes according to consensus CDF and the area under CDF curve. Thereafter, Kaplan‐Meier analysis represented the prognosis procedure of each subtype, and ‘survival’ package was used to draw the survival curve. ‘stats’ package was used for principal component analysis (PCA).

### 2.4 Gene function analysis

We conducted infiltration of immune cells analysis and single‐sample gene set enrichment analysis (ssGSEA) in pyroptosis‐related genes to explore the functional differences between the subtypes. We calculated the infiltration score of immune cells and compared among different subtypes by ‘GSEABase’ package. The ‘TIMER 1.0’^20^ database was used to examine the relationships between pyroptosis‐related gene expression and the level of infiltration of six immune cells in LGG and GBM samples. ‘GSVA’ package was used for GSVA analysis to acquire the difference in signalling pathway expressions between each two subtypes. In the drug sensitivity analysis, we downloaded the drug activity data of 60 cancer cell lines from the ‘CellMiner’^21^ database and created a scatter plot to calculate the Spearman coefficients between gene expression and drug sensitivity with ‘impute’, ‘limma’, ‘ggplot2’ and ‘ggpubr’ packages in R.

### 2.5 Derivation and validation of a prognostic model

We extracted data from TCGA as a training cohort for prognostic model derivation and extracted data from CGGA as test cohort to evaluate the performance of our prediction model. We first used Lasso and Cox regression to estimate the correlation between pyroptosis‐related genes and surviving status (‘glmnet’ and ‘survival’ package). In Lasso regression, we performed cross‐validation for 1000 times to acquire a robust model. There were 8 genes related to survival according to the penalty parameter (λ), and they were used to construct a multivariate Cox regression model. We further selected optimal gene collections using forward‐backward algorithm in Cox regression and applied them into survival prediction. In addition, the Kaplan‐Meier method was introduced to generate survival curves in the training and test sets. Then, the receiver operating curves (ROC) of 1, 3, and 5 years were used to test the predictive ability of the prognostic model (‘survivalROC’ package).

### 2.6 Statistical analysis

**FIGURE 1:**
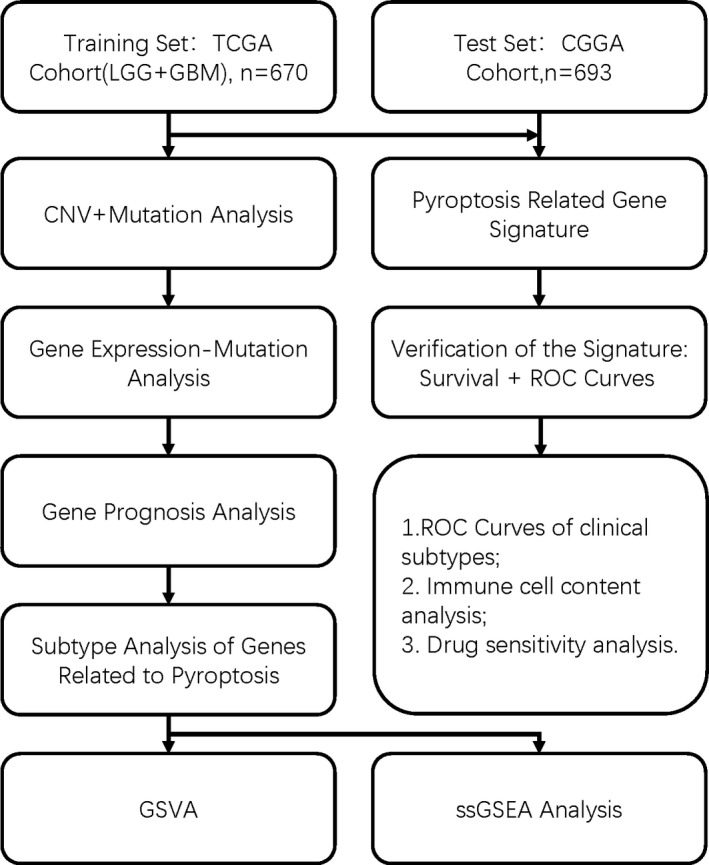
Flow chart of our research

t Test was applied to compare the gene expression levels between normal and mutated samples. For Kaplan‐Meier curves, we used Log‐rank test. To assess the independent prognostic value of the risk model, we used multivariate Cox regression models. All statistical analyses were accomplished with R software (v4.0.2). The overall flow diagram is shown in Figure 1.

## 3 RESULT

### 3.1 Landscape of genetic variation of pyroptosis gene in glioma

A total of 33 pyroptosis genes were finally identified in paper review.^18^, ^22^, ^23^, ^24^ We first summarized the incidence of copy number variations (CNV) of the pyroptosis‐related genes in glioma in the TCGA cohort and found that the incidence of loss of pyroptosis genes was greater than the incidence of gains in glioma samples. Among them, NLRP7, NLRP2, NLRP6 and CASP3 had a higher incidence of loss (Figure S1A, S1B). Further analyses revealed the mutation characteristics of the pyroptosis genes in each glioma sample (Figure S1C, S1D), which indicated that the mutation rate of all pyroptosis genes is very low (less than or equal to 3%), indicating these genes are more conservative. We next performed the association analysis on the mutations of the pyroptosis gene and found the connection of the mutations and the downstream gene expression (Figure S2). Among the pyroptosis genes, NLRP3, PLCG1 and CASP1 are the key genes. Interestingly, we found that NLRP3 mutation tumours showed high expression of CASP6, GSDME, GSDMD, GPX4 and CASP3, and PLCG1 mutation tumours showed a high expression of PJVK, AIM2 and GSDMB and these genes were also regulated by other gene mutations, while CASP1 mutation tumours showed a low expression of PLCG1, NOD1, SCAF11, TIRAP and high expression IL6. Figure S3 showed a summary of mutations in the pyroptosis genes resulting in a significant change in the expression of other pyroptosis genes.

### 3.2 Correlation between pyroptosis gene expression and prognosis

**FIGURE 2:**
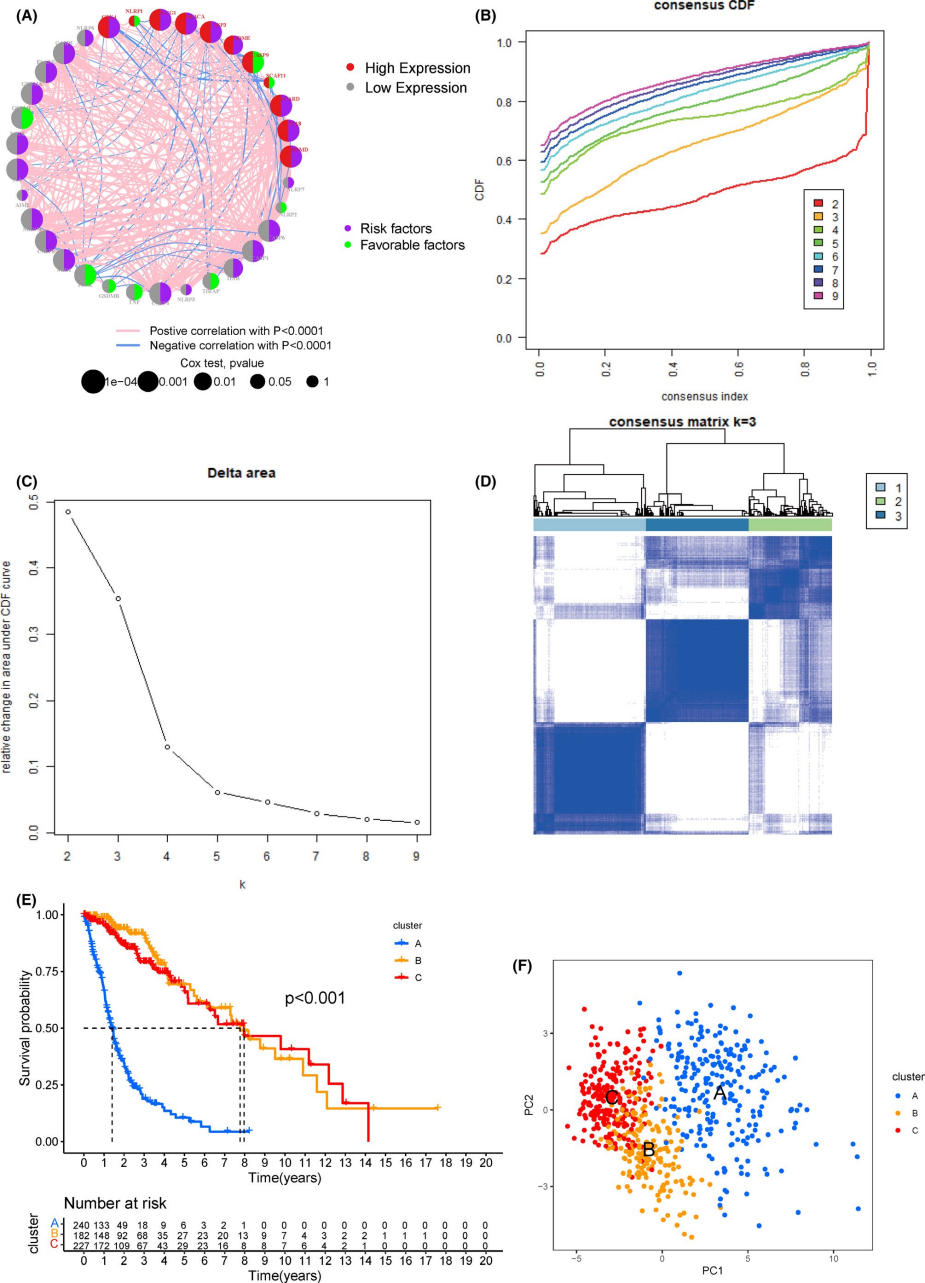
A. Circos graph for univariate cox regression analysis, which represents the correlation of pyroptosis gene expression (grey: low expression, FPKM<5; red: high expression, FPKM>5) and prognosis of glioma cancer (purple: risk factor; green: favourable factors) in the TCGA cohort (P values for cox test: 1e‐ 04 to 1, and bigger bubbles mean the correlations were of more statistical significance). B. Census CDF curves for the TCGA cohort. C. Delta area under CDF curve shows the change of accumulative risk along with the increased consensus clustering matrixes and demonstrated that three clusters were optimal (k=3). D. 760 patients were grouped into three clusters according to the consensus clustering matrix (k=3). E. Kaplan‐Meier curves for comparison of the three clusters, and time‐dependent numbers at risk in three clusters. F. PCA plots for three clusters in the TCGA cohort

To explore the relationship between pyroptosis and prognosis of glioma patients, we conducted a comprehensive analysis. The comprehensive landscape of 33 pyroptosis genes interactions, expression and their prognostic significance for glioma patients was depicted in Figure 2A. We found that the up/down‐regulation of most pyroptosis genes had significant impacts on prognosis, and most of them are risk factors. Next, we used the consensus clustering method to explore glioma subtypes based on the expression of pyroptosis genes. After evaluating the relative change in area under the cumulative distribution function (CDF) for each category number k compared with k ‐ 1, we found that when choosing a 3‐cluster solution (k = 3), the area came to the maximum increasing speed. When increasing the number of clusters from 2 to 8, we found that the cumulative distribution function value was close to the maximum increasing speed when k = 3 (Figure 2B). When the number of total subtypes increasing, the area under the CDF curve rises less (Figure 2C). For different subtypes, within‐group correlations proved strong while between‐group correlations proved moderate (Figure 2D). Therefore, it is reasonable to classify TCGA glioma samples based on the expression of pyroptosis genes. At the same time, prognostic analysis for the three subtypes (Figure 2E) revealed that the prognosis of subtype A was worse than the other two types (*p *< 0.001), and no significant difference was observed between subtype B and subtype C. In addition, principal component analysis (PCA) shows that the three subtypes can be distinguished well in a two‐dimensional space (Figure 2F).

**FIGURE 3:**
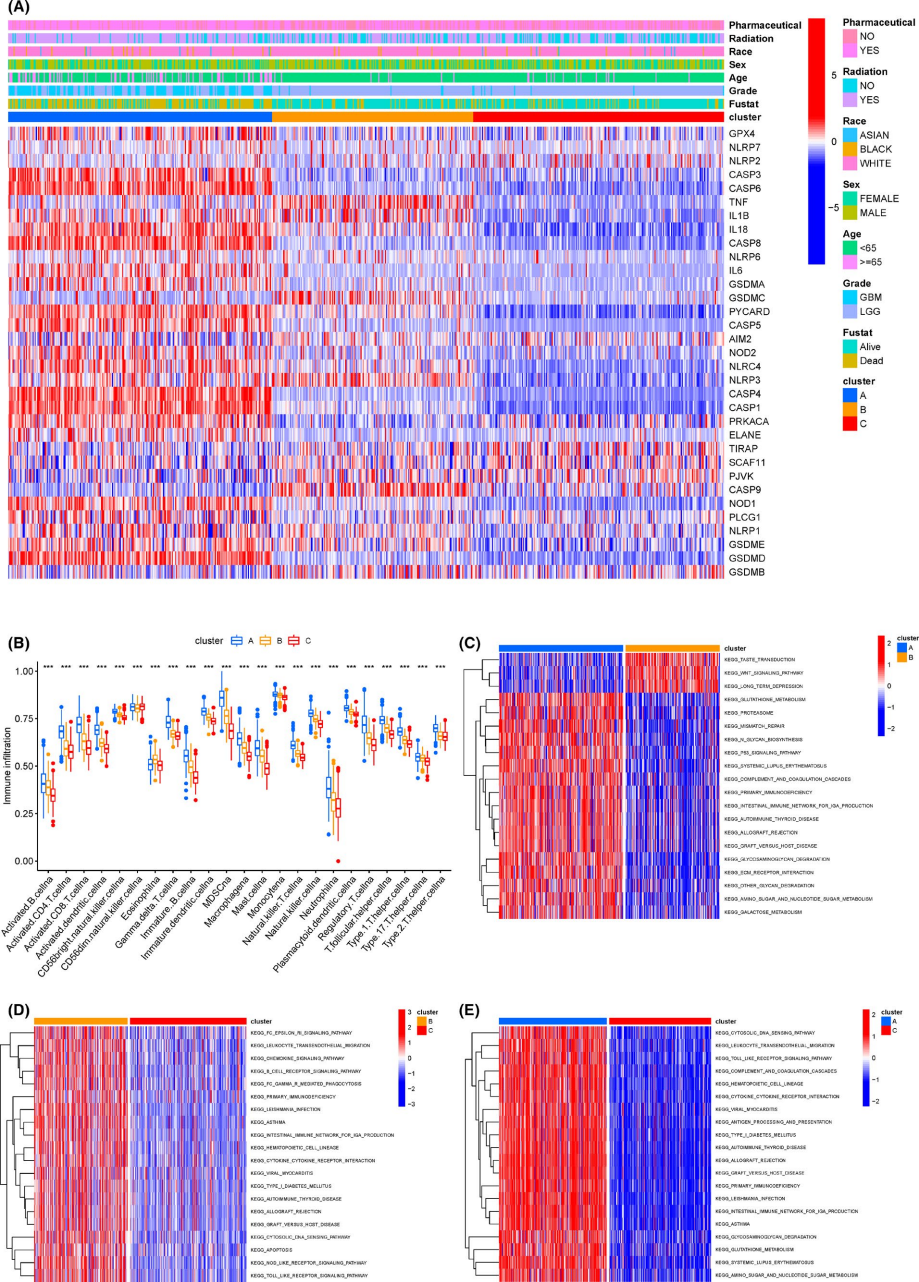
A. Heatmap for the connections between clinicopathologic features and the three gene clusters (from blue to red means increased gene expression). B. Comparison of the immune infiltration among three clusters in the TCGA cohort. C‐E. Pair wised GSVA analysis between each two clusters

To discover the phenotypic and genomic differences of the three subtypes, we plotted heat maps about the basic characteristics of patients in the TCGA cohort and the differences in pyroptosis gene expression (Figure 3A). We found that subtype A had more dead samples, and GBM‐type glioma (a lower degree of differentiation and a higher degree of malignancy glioma subtype) accounted for the largest proportion. Regarding the expression of pyroptosis genes, the expression of pyroptosis genes in the subtype A with the worst prognosis level was extensively up‐regulated, that of subtype C, however, were down‐regulated, and that of subtype B came in the middle.

### 3.3 Pyroptosis‐related gene function analysis

Previous studies demonstrated that pyroptosis was closely related to immunity; to explore the role of pyroptosis in immunity of glioma, we performed ssGSEA analysis on the TCGA cohort to compare the immune activity between subtypes (Figure 3B). The results showed that among the 23 immune cell enrichment scores, the high‐risk subtype (subtype A) had a higher level of infiltration of immune cell, especially activated CD4T cells, immature B cells, neutrophilia, etc. In addition, compared with the subtype C, the enrichment scores of most immune cells were also higher in the subtype B.

To investigate the biological behaviours among these distinct subtypes of glioma, we also performed GSVA analysis to compare the differences in the expression of cell function and signal pathway between each two subtypes. Cluster‐A was markedly enriched in signal pathways such as P53 signalling, intestinal immune network for IGA and immune rejection signalling pathway (Figure 3C). Cluster‐B presented enrichment pathways associated with immune‐related signal pathways such as B‐cell receptor, cytophagy and chemokine (Figure 3D), while cluster‐C was mainly down‐regulated in signal pathways such as apoptosis, immune‐related signalling pathways (Figure 3E). Therefore, the classification of glioma based on pyroptosis mainly had differential expression in signal pathways including immunity and inflammation.

### 3.4 Development of a prognostic model for glioma based on pyroptosis gene

**FIGURE 4:**
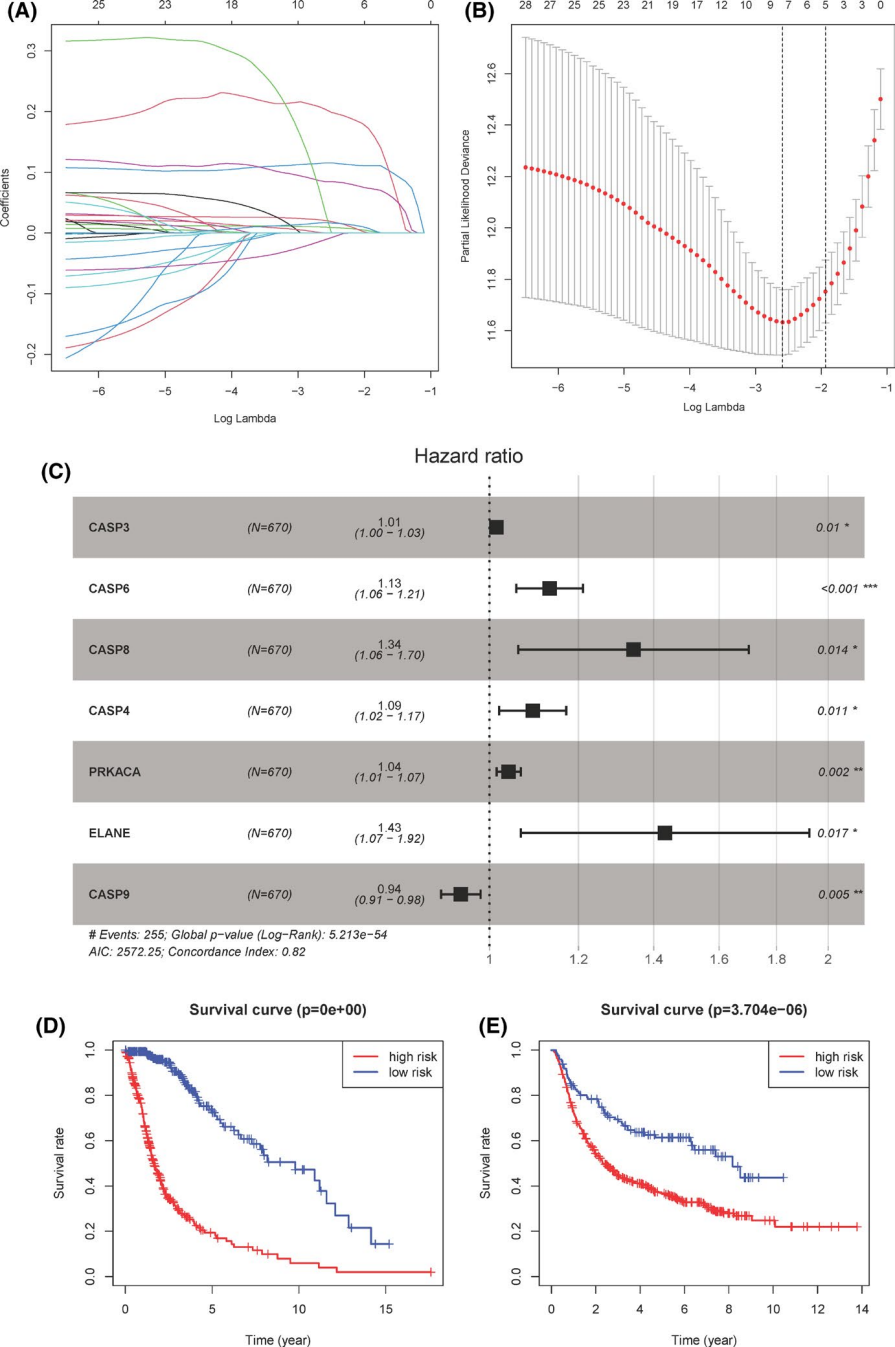
A. Lasso regression for the 33 pyroptosis‐related genes. B. Cross‐validation for tuning the parameter selection in the LASSO regression. C. Forest plot for hazard ratios of 7 pyroptosis‐related genes (*P<0.05, **P<.01, ***P<0.001). D‐E. Kaplan‐Meier curves for comparison of the OS in training set (TCGA) and validation set (CGGA)

**FIGURE 5:**
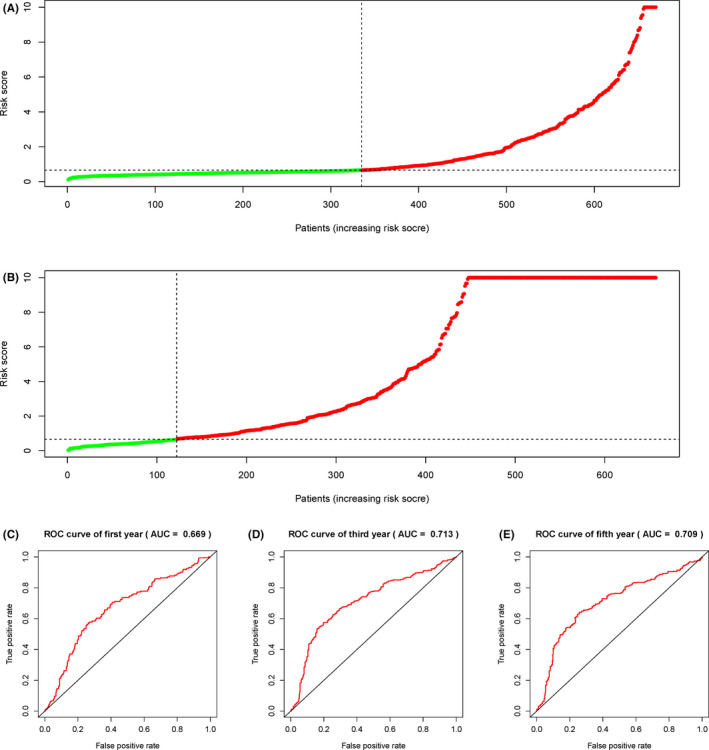
A. Distribution of patients in the TCGA cohort based on the risk score. B. Distribution of patients in the CGGA cohort based on the median risk score in the TCGA cohort. C‐E. Time‐dependent ROC curves for 1‐year survival, 3‐year survival and 5‐year survival in the CGGA cohort

The summary of clinical characteristics of TCGA and CGGA cohorts was shown in Table 1. Considering the importance of pyroptosis genes in cancer, we constructed a prognostic model using 670 glioma samples from TCGA. We first used the least absolute contraction and selection operator (LASSO) regression analysis to determine the 8 gene signatures according to the optimal λ value (Figure 4A, B) and further used the multivariate cox regression analysis to determine the optimal 7 genes and risk calculation formula (Figure 4C, Table 2). The risk score was expressed as: Risk Score = (0.014*CASP3 exp.) + (0.123*CASP6 exp.) + (0.295*CASP8 exp.) + (0.089*CASP4 exp.) + (0.039*PRKACA exp.) + (0.359*ELANE exp.) + (−0.059*CASP9 exp.). After completing the development of the glioma prognostic model, we used 693 glioma samples from CGGA as a test cohort to evaluate the performance of the prognostic model. According to the median of the risk scores in the TCGA cohort as the cut‐off value, the TCGA and CGGA samples were divided into low‐risk and high‐risk subgroups. Next, Kaplan‐Meier curves were used for survival analysis. We found that the high‐risk score had less survival time in both TCGA and CGGA data set (*p *< 0.001) (Figure 4D, E). In addition, Figure 5A and B showed the risk score distribution of the prognostic model on the TCGA and CGGA samples. Finally, the prognostic model achieved good predictive performance on CGGA test cohort, and the AUC values in the 1‐year survival rate (Figure 5C), 3‐year survival rate (Figure 5D) and 5‐year survival rate (Figure 5E) were 0.669, 0.713 and 0.709 respectively; while the AUC values were 0.867, 0.892 and 0.836 respectively on the TCGA samples (Figure S3).

**TABLE 1:** The clinical information of TCGA and CGGA

	TCGA (Training set)	CGGA (Test set)
N	670	693
Age	57.0±15.8	48.3±12.4
State (%)		
Alive	296 (44.2)	296 (42.7)
Dead	374 (55.8)	397 (57.3)
Gender (%)		
Male	393 (58.7)	398 (57.4)
Female	277 (41.3)	295 (42.6)
OS (days)	712.0±795.4	1199.2±998.1
IDH mutation status (%)		
Wild type	‐	286 (41.3)
Mutant	‐	356 (51.4)
Unknown	‐	51 (7.3)
1p19q codeletion status		
Codel	‐	145 (20.9)
Non‐codel	‐	478 (69.0)
Unknown	‐	70 (10.1)
MGMTp methylation status		
Methylated	‐	315 (45.5)
Un‐methylated	‐	227 (32.8)
Unknown	‐	151 (21.7)
Chemotherapy (%)		
Yes	499 (74.5)	486 (70.1)
No	171 (25.5)	207 (29.9)
Radiotherapy (%)		
Yes	487 (72.7)	510 (73.6)
No	183 (27.3)	183 (26.4)
Race (%)		
White	620 (92.5)	0 (0.0)
Asian	11 (1.6)	693 (100.0)
Black	39 (5.9)	0 (0.0)
		

**TABLE 2:** Parameters of the pyroptosis gene signature

id	coef	HR	HR.95L	HR.95H	P value
CASP3	0.014	1.015	1.003	1.026	0.010
CASP6	0.123	1.131	1.056	1.211	0.000
CASP8	0.295	1.343	1.061	1.700	0.014
CASP4	0.089	1.093	1.020	1.170	0.011
PRKACA	0.039	1.040	1.015	1.066	0.002
ELANE	0.359	1.432	1.066	1.924	0.017
CASP9	‐0.059	0.943	0.906	0.982	0.005

**FIGURE 6:**
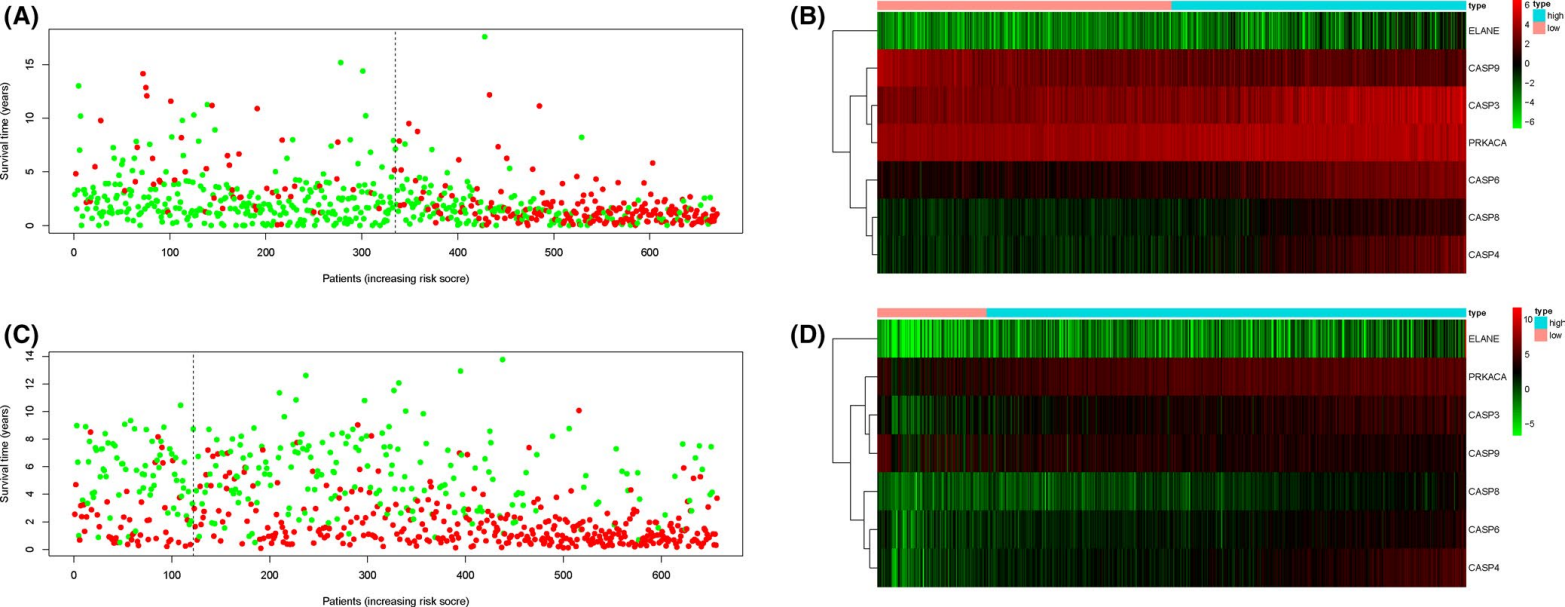
A. The survival status for each patient in the TCGA cohort (low‐risk population: on the left side of the dotted line; high‐risk population: on the right side of the dotted line). B. Heatmap (green: low expression; red: high expression) for the connections between clinicopathologic features and the risk groups (high, brilliant blue; low, red) in the TCGA cohort. C. The survival status for each patient in the CCGA cohort (low‐risk population: on the left side of the dotted line; high‐risk population: on the right side of the dotted line). D. Heatmap (green: low expression; red: high expression) for the connections between clinicopathologic features and the risk groups (high, brilliant blue; low, red) in the CCGA cohort

Furthermore, we observed the trend of survival status and pyroptosis gene expression by risk scores on the TCGA (Figure 6A) and CGGA data sets (Figure 6C) respectively by drawing the risk score‐survival time scatter plot, and the survival status of the sample is positively correlated with the risk score. The proportion of dead samples was larger in high‐risk score subgroup than in low‐risk score subgroup. At the same time, we found that the expression of pyroptosis genes was significantly different in the high/low‐risk score subgroup in the TCGA and CGGA data set, including the high expression of CASP3, CASP6, CASP8, CASP4 and ELANE in the high‐risk group and the low expression of CASP9 in the high‐risk group (Figure 6B, D), and the results have the same conclusions as Table 2.

We also used the ‘TIMER’ database to investigate the relationships between pyroptosis‐related gene expression in our model and immune activities in B cells, CD8+ T cells, CD4+ T cells, macrophages, neutrophils and dendritic cells (Figure S5). We found that it has significantly positive correlation between the expression of CASP4, CASP6, and CASP8 and the immune cell, such as B cells, CD4+ T cells, macrophages, neutrophils, and dendritic cells (partial correlation >0.48 and *p* < 0.001) in LGG subtypes, which indicated CASP4, CASP6 and CASP8 might be key genes involved in tumour immunity in pyroptosis.

We examined the pyroptosis gene expression‐drug activity correlation of 60 tumour cell lines using the ‘CellMiner’ database to evaluate the potential of pyroptosis genes as antitumour drug targets (Figure S6) in pan‐cancer. The results show that expression of ELANE, PRKACA, CASP3, CASP9 and CASP6 was related to the efficacy of various anticancer drugs, indicating that pyroptosis genes may participate in pan‐cancer signalling pathways and affect the efficacy of anticancer drugs.

In addition, we divided the CGGA test set results according to clinical subtypes, such as gender, age and radiotherapy/chemotherapy status. The AUC of 1/3/5‐year survival rate of the subgroups divided by gender or age in the test set was all greater than 0.65 (Figure S7). Moreover, the 3/5‐year survival rate of the female or >60‐year‐old subgroup was better than other subgroup (AUC value greater than 0.70) (Figure S8). The prediction results of 1/3/5‐year survival rate of clinical subgroups based on radiotherapy/chemotherapy status indicate that our prognostic model showed good prediction performance (AUC value greater than 0.65) (Figure S8). The above results demonstrate the robustness of our prognostic model and its excellent predictive ability for the rare clinical subgroup (>60 years old). In summary, our prognostic model can also maintain consistent predictive performance for samples that implement different clinical treatment plans, which reflects the clinical application value of the prognostic model of glioma based on pyroptosis.

## 4 DISCUSSION

In this study, we first analysed the mutations and variations of 33 known pyroptosis‐related genes in glioma samples and found that these genes were conservative and stable expression. After that, we determined the significant correlation of these genes to prognosis through a single‐gene prognostic network. To further evaluate the prognostic value of these pyroptosis‐related regulatory factors, we performed a consensus clustering of glioma samples to determine three subtypes of glioma based on pyroptosis. Survival analysis showed a significant difference among three subgroup of glioma. Meanwhile, GSVA and ssGSEA analysis showed that the pyroptosis gene plays an important role on tumour immune‐related signal pathways to participate in the prognostic process of tumours. Then, the drug sensitivity analysis demonstrated multiple associations between pyroptosis gene expressions and antitumour drug activities in pan‐cancer cell lines. Through LASSO analysis and multivariate Cox analysis, we constructed a glioma prognostic model containing 7 pyroptosis‐related genes and validated its performance both in the overall external test cohort and different clinical subgroups, by which the glioma patients can be meaningfully distinct high‐risk and low‐risk groups.

Pyroptosis is a newly discovered method of programmed cell death. Several studies have shown that pyroptosis can participate in and affect tumour immunity and treatment processes. Pyroptosis not only can release inflammatory substances such as IL‐1 and IL‐18 to promote the development and progression of tumours, but also it has become a new therapeutic mechanism to induce cancer cells to initiate the process of pyroptosis by combining targeted drugs with the pyroptosis‐related proteins of cancer cells.^25^, ^26^ Glioma is the intracranial tumour with the highest incidence. However, previous studies did not involve the influence of pyroptosis in the development of glioma and its effect on the prognosis of patients.

In this study, we observed 7 pyroptosis‐related genes that are closely related to the prognosis of glioma through bioinformatics analysis and prognostic analysis. They are CASP3, CASP4, CASP6, CASP8, CASP9, PRKACA and ELANE. Caspases‐3 (CASP3) is one of the key factors of cell apoptosis. At the same time, a study^27^ has shown that in addition to participating in cell apoptosis, activation of CASP3 can also induce pyroptosis in cancer cells and normal cells expressing GSDME. Zhang et al.^17^ found that in lung cancer cells, cisplatin and paclitaxel can activate CASP3 to promote the production of N‐terminal fragments of GSDME and cause pyroptosis; caspases‐4 (CASP4) is involved in another pathway of pyroptosis. GSDMD is cleaved specifically to initiate pyroptosis by CASP4.^11^ The pyroptosis pathway induced by CASP4 in tumour cells has also been confirmed by many studies^28^; caspases‐6 (CASP6) is a key regulator of innate immunity, inflammasome activation and host defence. Previous studies mostly thought that CASP6 is the executor of apoptosis. Moreover, Zheng et al.^29^ found that the pyroptosis phenomenon was reduced in CASP6 knockout macrophages infected with influenza A virus (IAV), including the lysis of caspase‐1 and IL‐ The release of 1β and IL‐18 is reduced, so it can be considered that CASP6 is a potential regulator involved in pyroptosis; caspases‐8 (CASP8) mainly induces exogenous cell apoptosis^30^ and can inhibit necroptosis mediated by RIPK3 and MLKL. At the same time, Fritsch et al.^31^ found that the inactivation of CASP8 in the later stage of the mouse embryo will lead to cell death different from apoptosis and further found that the expression of CASP8 leads to the activation of caspase‐1 and the secretion of IL‐1β, thereby participating in pyroptosis. Caspases‐9 (CASP9) is involved in apoptosis and autophagy. An et al.^32^ reported that CASP9 can positively regulate autophagy by maintaining the maintenance of mitochondrial homeostasis. Mitochondrial damage can activate NLRP3 inflammasome and cause further mitochondrial damage and IL‐1β‐dependent inflammation, thereby affecting pyroptosis.^33^ Therefore, CASP9 seems to be able to inhibit pyroptosis. Protein kinase cAMP‐activated catalytic subunit alpha (PRKACA) is closely related to tumour progression activated by cAMP. A study^34^ showed that PRKACA mutations were found in approximately 37–66% of adrenal adenoma samples in patients with Cushing's syndrome. In addition, Moody et al.^35^ found that the expression of PRKACA can cause the inactivation of the pro‐apoptotic protein BAD in breast cancer cells to initiate the BCL‐XL/BCL‐2 anti‐apoptotic pathway. Elastase (ELANE) is a key component of the innate immune system, which mediates the elimination of pathogens through a variety of mechanisms. In addition, Kambara et al.^36^ found that the cleavage and activation of GSDMD in neutrophils are mediated by ELANE, which can cleave GSDMD upstream of the caspase cleavage site to generate smaller but still biologically active GSDMD‐eNT fragments.

As tumours develop, multiple modes of cell death may interact. In this study, PRKACA, CASP3, CASP6 and CASP8 are also the main executors of apoptosis, and the characteristic of apoptosis is that the plasma membrane of the cell is intact, the contents are not released, and it does not directly cause inflammation.^37^ In addition, we analysed differences in the level of immune infiltration and signal pathway expression between different subtypes through GSVA and ssGSEA, and proved that the differential expression of pyroptosis mainly affects immune and inflammation‐related functions, indicating that pyroptosis also affects the tumour immune microenvironment composition.

## 5 CONCLUSION

In this study, we set up an effective glioma prognosis model based on pyroptosis‐related genes. The AUC values of this model in test set can achieve 0.669, 0.713 and 0.709 in 1, 3 and 5 years, respectively. The genes in the model are closely related to immune infiltration and drug sensitivity. This model can provide a reference for clinical decision‐making. This study still has some limitations. First, the results obtained based on the RNAseq data set still need to be verified by cell experiments and prospective clinical trials. Secondly, how to interact between pyroptosis genes still needs further study.

## CONFLICT OF INTEREST STATEMENT

The authors declare no conflicts of interest.

## AUTHOR CONTRIBUTION

**Bo Chao:** Conceptualization (lead); Investigation (equal); Methodology (equal); Visualization (equal); Writing‐original draft (equal). **Fenjun Jiang:** Investigation (equal); Methodology (equal); Software (lead); Visualization (equal); Writing‐original draft (equal). **Huiru Bai:** Investigation (supporting); Methodology (supporting). **Peipei Meng:** Investigation (supporting); Visualization (supporting). **Lu Wang:** Funding acquisition (supporting); Writing‐review & editing (supporting). **Fei Wang:** Funding acquisition (lead); Writing‐original draft (lead); Writing‐review & editing (lead).

## Supporting information

Fig S1

Fig S2

Fig S3

Fig S4

Fig S5

Fig S6

Fig S7

Fig S8

## Data Availability

All the data in our study can be accessed from the online database.
