# Supplementary figures and images for: Predicting the prognosis of glioma by pyroptosis‐related signature
Source: J Cell Mol Med. 2021 Nov 23;26(1):133–43. doi: 10.1111/jcmm.17061 (PMC8742236; doi:10.1111/jcmm.17061)

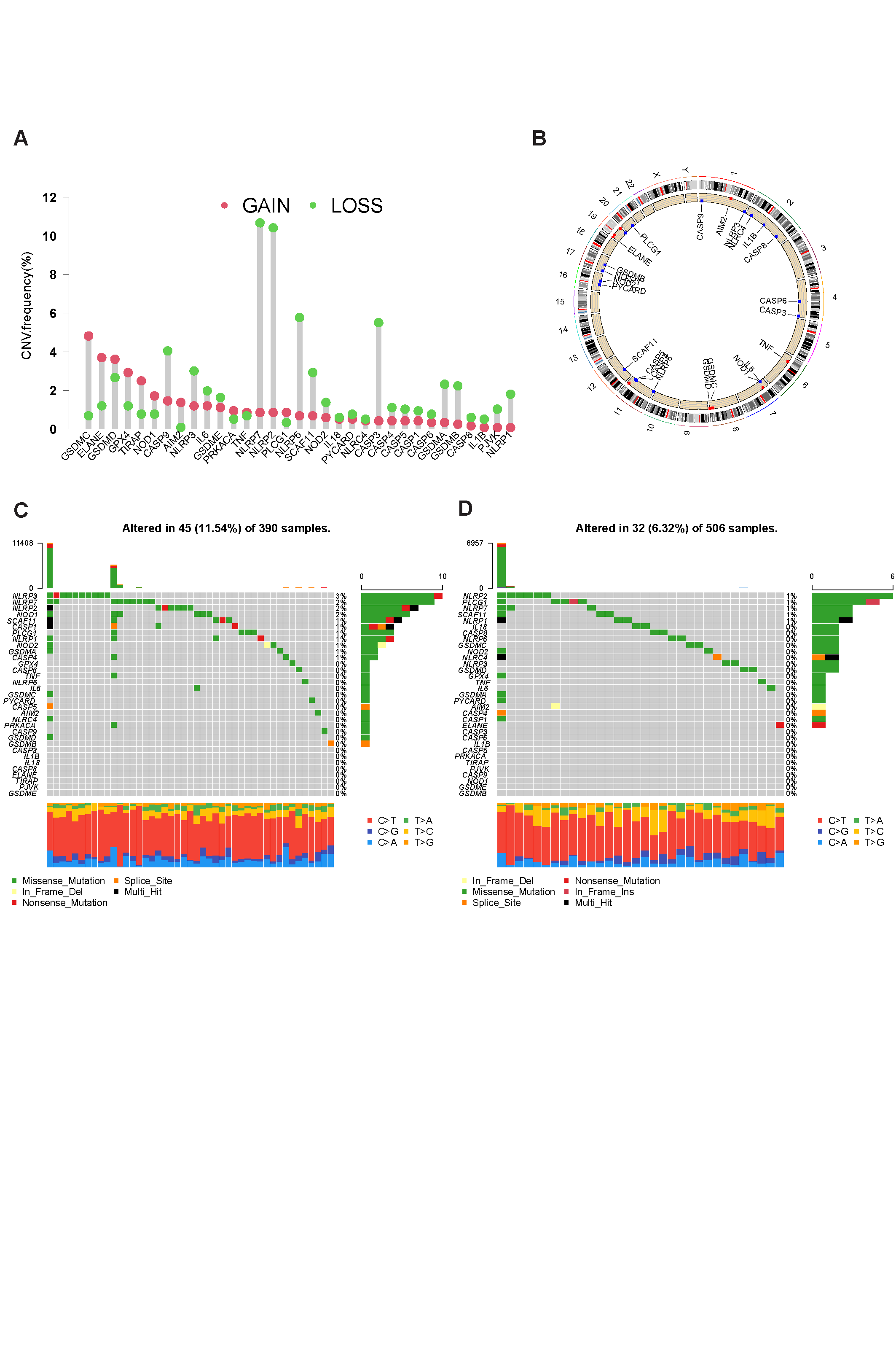

Supplement: Supplementary file 1 — Fig S1 [file JCMM-26-133-s005.tiff]

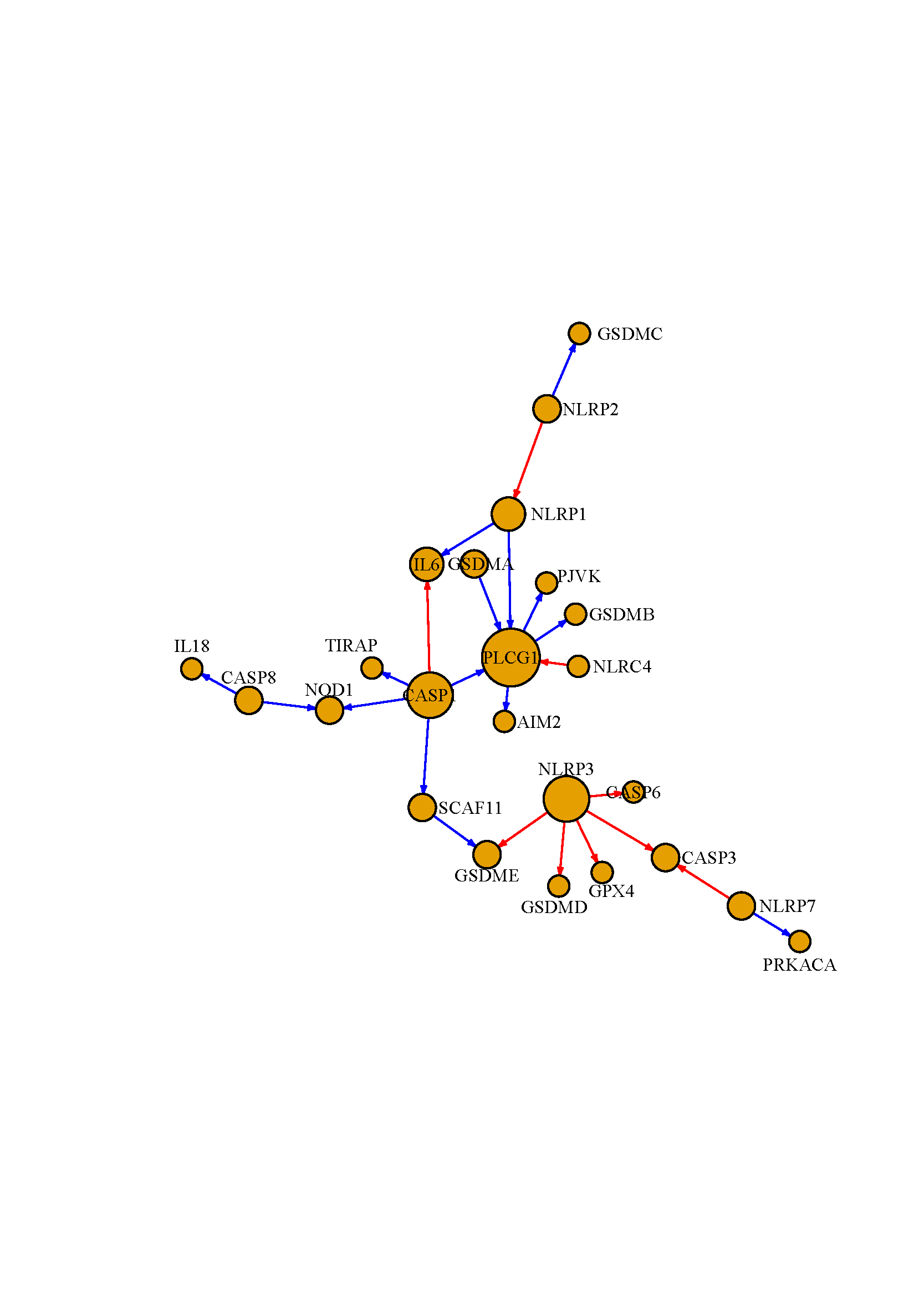

Supplement: Supplementary file 2 — Fig S2 [file JCMM-26-133-s003.tiff]

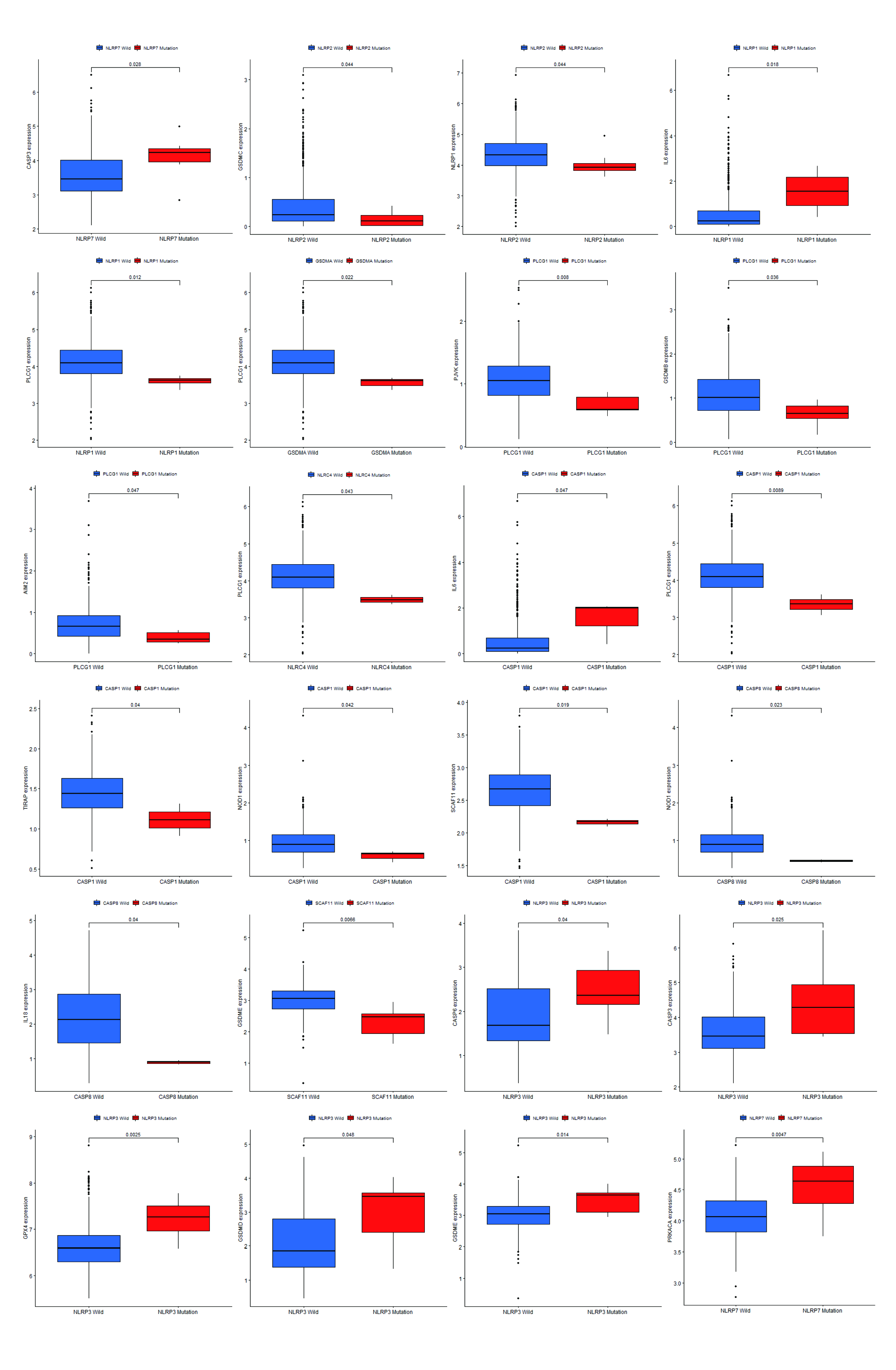

Supplement: Supplementary file 3 — Fig S3 [file JCMM-26-133-s002.tiff]

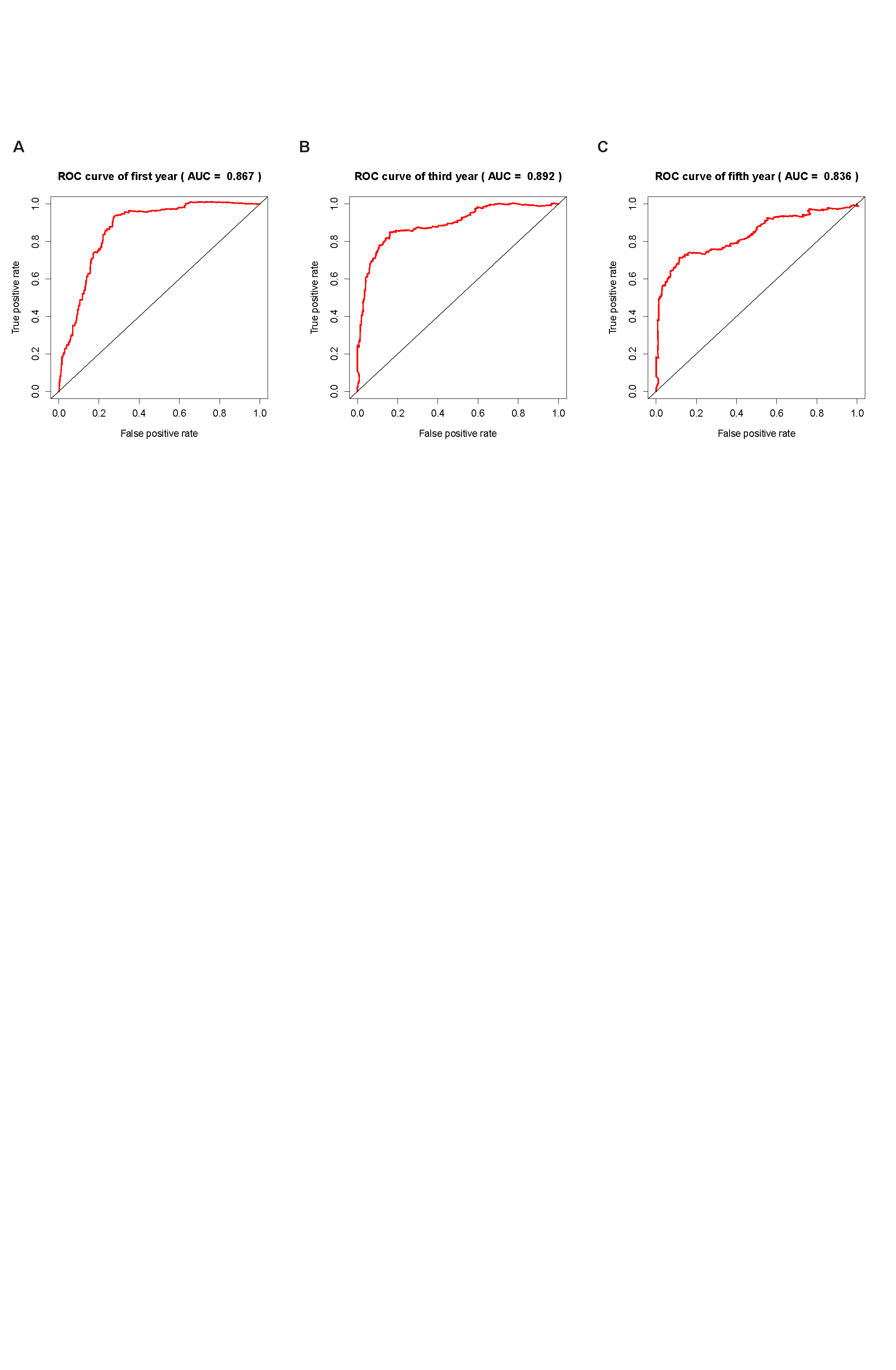

Supplement: Supplementary file 4 — Fig S4 [file JCMM-26-133-s008.tiff]

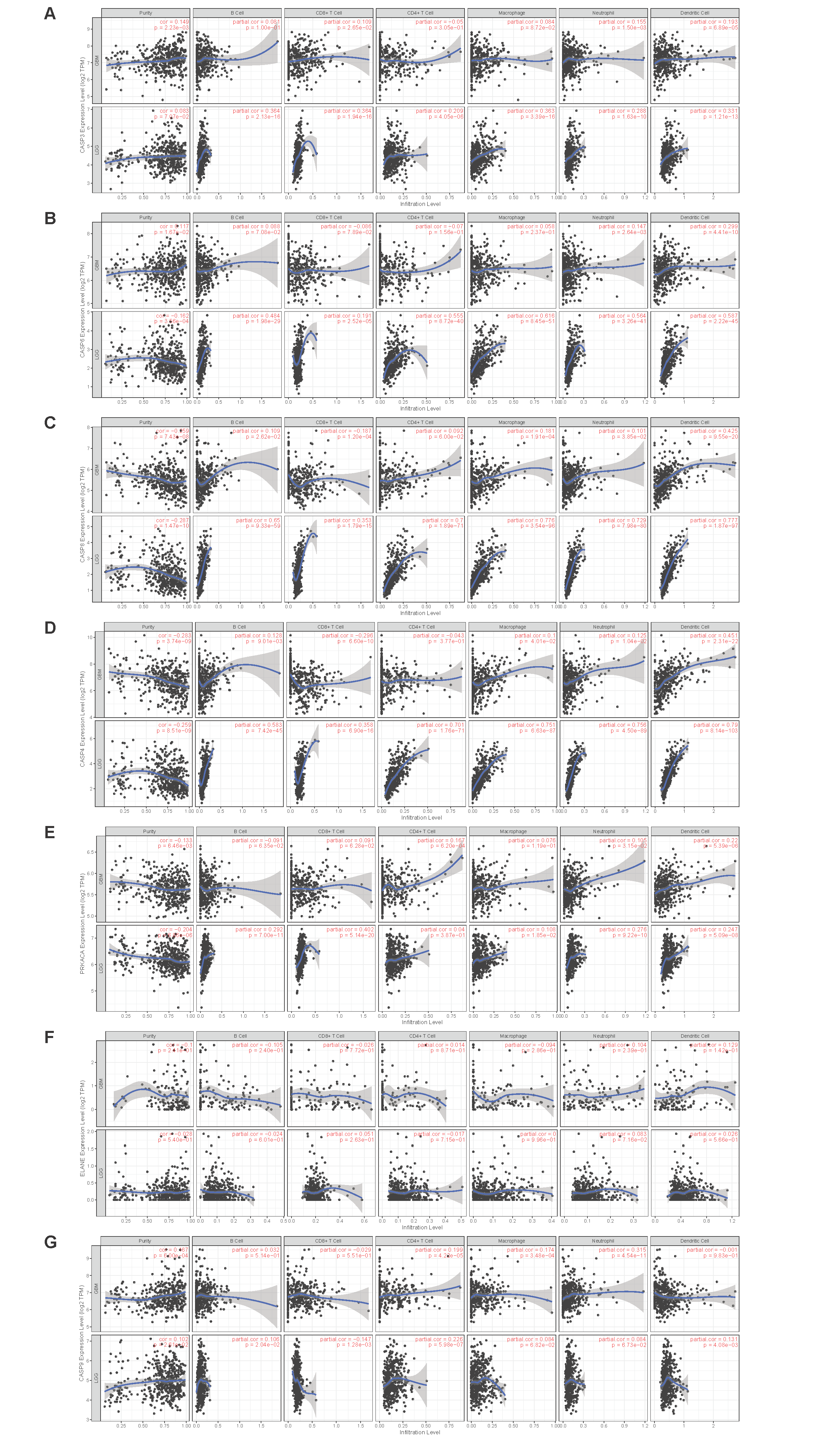

Supplement: Supplementary file 5 — Fig S5 [file JCMM-26-133-s007.tiff]

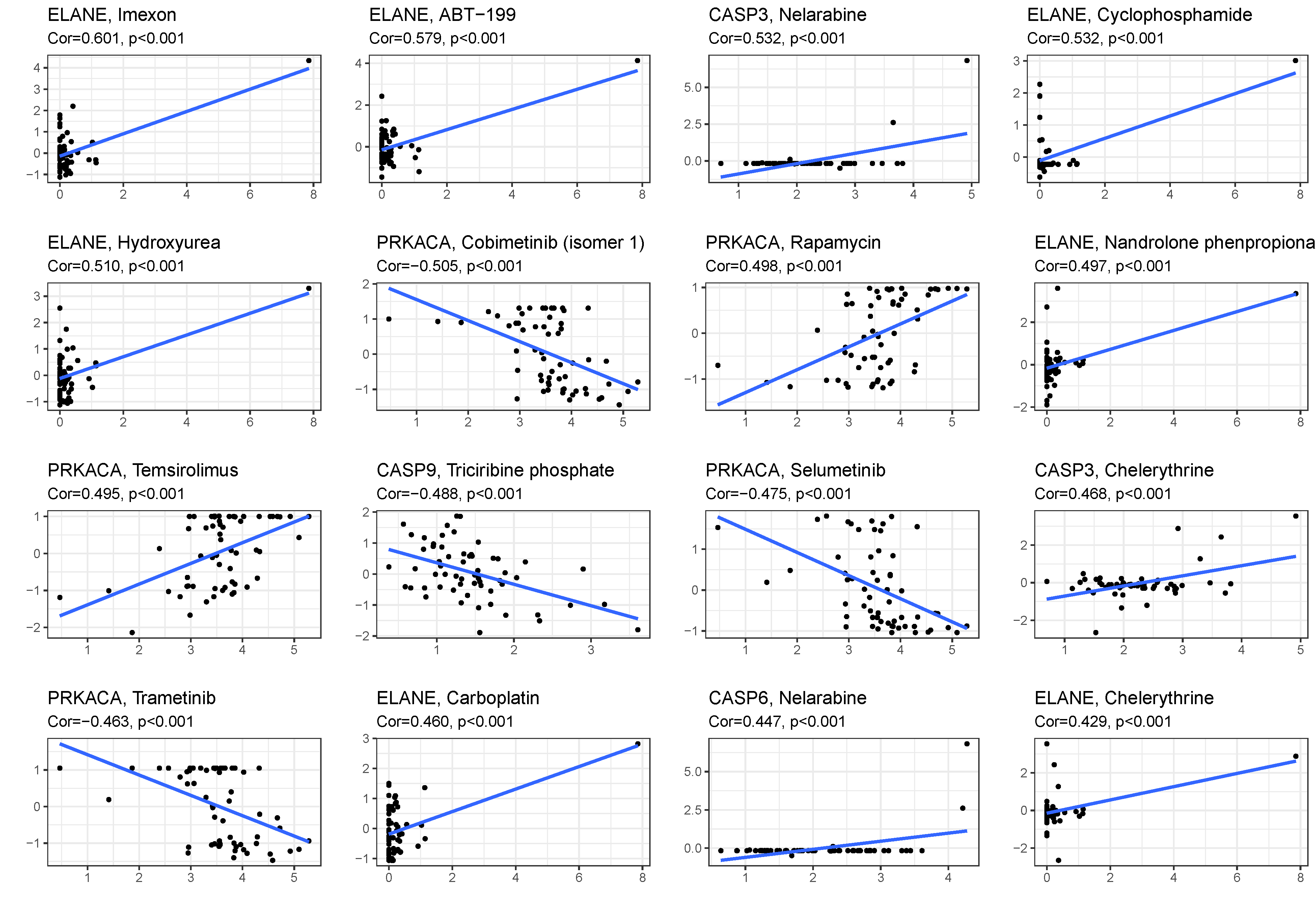

Supplement: Supplementary file 6 — Fig S6 [file JCMM-26-133-s006.tiff]

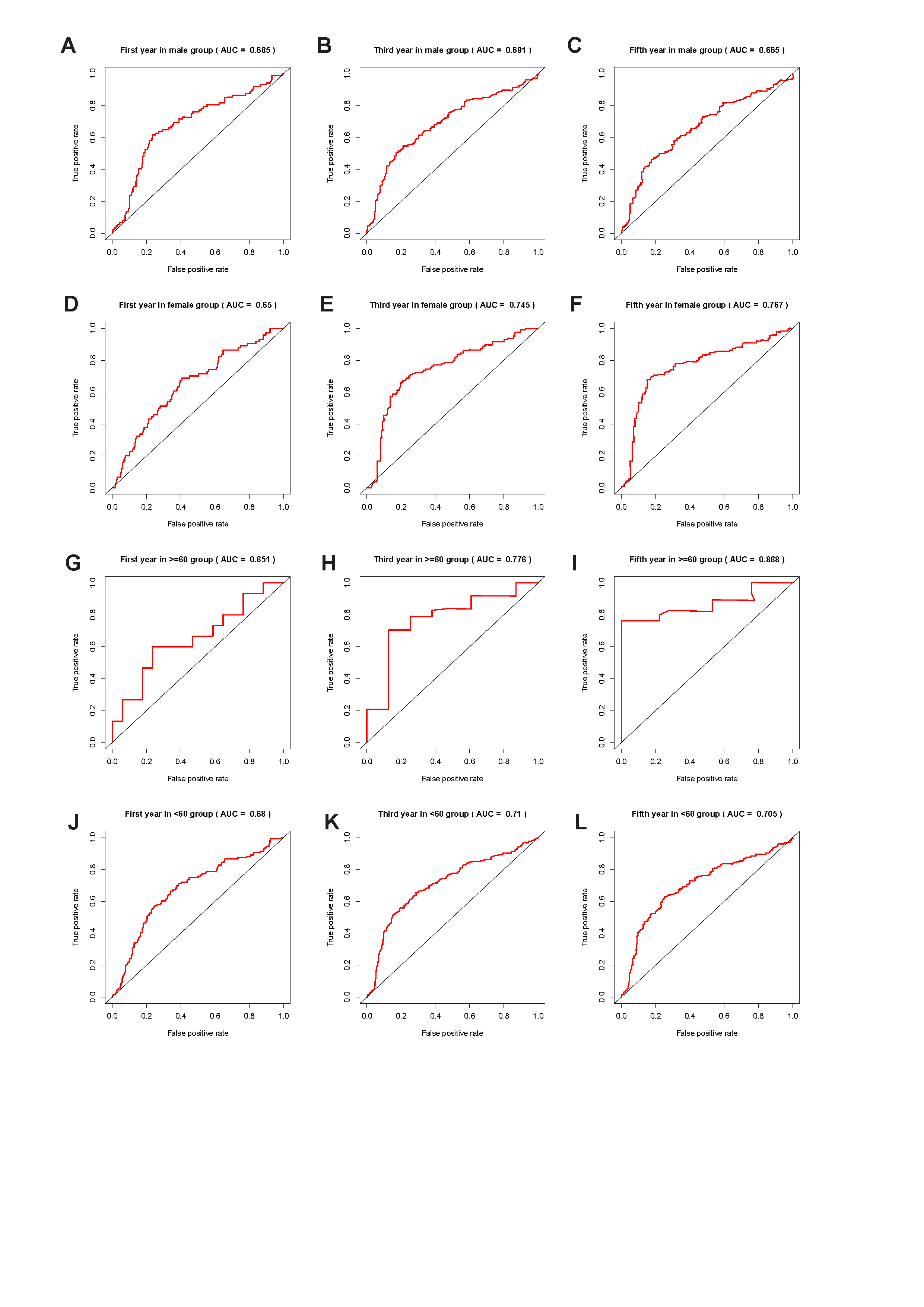

Supplement: Supplementary file 7 — Fig S7 [file JCMM-26-133-s004.tiff]

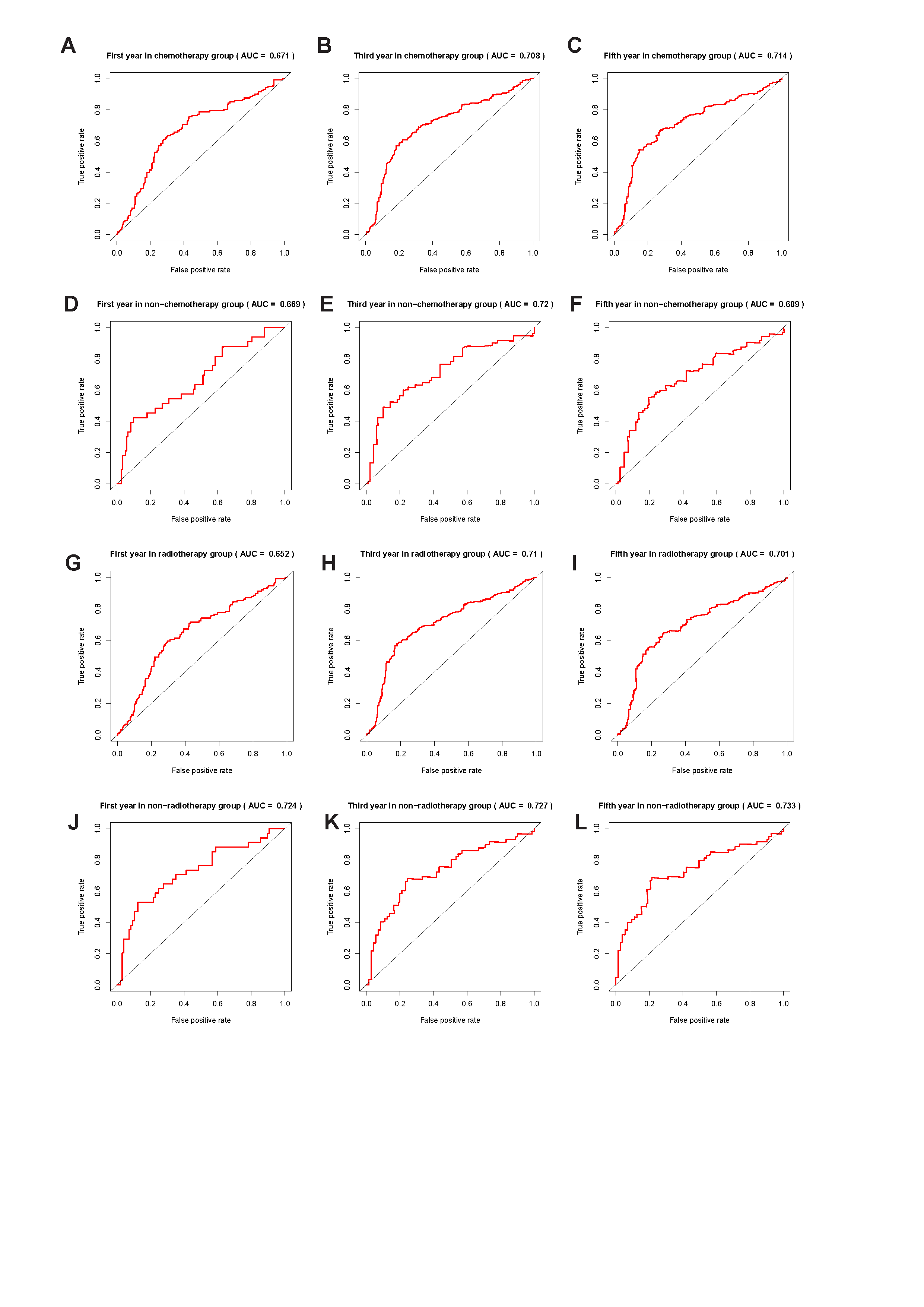

Supplement: Supplementary file 8 — Fig S8 [file JCMM-26-133-s001.tiff]
